# Exploring Microbial Influence on Flavor Development during Coffee Processing in Humid Subtropical Climate through Metagenetic–Metabolomics Analysis

**DOI:** 10.3390/foods13121871

**Published:** 2024-06-14

**Authors:** Alexander da Silva Vale, Cecília Marques Tenório Pereira, Juliano De Dea Lindner, Luiz Roberto Saldanha Rodrigues, Nájua Kêmil El Kadri, Maria Giovana Binder Pagnoncelli, Satinder Kaur Brar, Carlos Ricardo Soccol, Gilberto Vinícius de Melo Pereira

**Affiliations:** 1 Department of Bioprocess Engineering and Biotechnology, Federal University of Paraná (UFPR), Curitiba 81530-900, PR, Brazilsoccol@ufpr.br (C.R.S.); 2 Department of Food Science and Technology, Federal University of Santa Catarina (UFSC), Florianópolis 88034-000, SC, Brazil; tenorioctn@gmail.com (C.M.T.P.); juliano.lindner@gmail.com (J.D.D.L.); 3 Graduate Program in Biotechnology, Federal Technological University of Paraná (UTFPR), Dois Vizinhos 85660-000, PR, Brazil; luiz@capricorniocoffees.com.br (L.R.S.R.); giobinder@gmail.com (M.G.B.P.); 4 Department of Civil Engineering, Lassonde School of Engineering, York University, North York, Toronto, ON M3J 1P3, Canada; satinder.brar@lassonde.yorku.ca

**Keywords:** coffee fermentation, cup quality, specialty coffee, SCA metrics, climate changes

## Abstract

Research into microbial interactions during coffee processing is essential for developing new methods that adapt to climate change and improve flavor, thus enhancing the resilience and quality of global coffee production. This study aimed to investigate how microbial communities interact and contribute to flavor development in coffee processing within humid subtropical climates. Employing Illumina sequencing for microbial dynamics analysis, and high-performance liquid chromatography (HPLC) integrated with gas chromatography–mass spectrometry (GC-MS) for metabolite assessment, the study revealed intricate microbial diversity and associated metabolic activities. Throughout the fermentation process, dominant microbial species included *Enterobacter*, *Erwinia*, *Kluyvera*, and *Pantoea* from the prokaryotic group, and *Fusarium*, *Cladosporium*, *Kurtzmaniella*, *Leptosphaerulina*, *Neonectria*, and *Penicillium* from the eukaryotic group. The key metabolites identified were ethanol, and lactic, acetic, and citric acids. Notably, the bacterial community plays a crucial role in flavor development by utilizing metabolic versatility to produce esters and alcohols, while plant-derived metabolites such as caffeine and linalool remain stable throughout the fermentation process. The undirected network analysis revealed 321 interactions among microbial species and key substances during the fermentation process, with *Enterobacter*, *Kluyvera*, and *Serratia* showing strong connections with sugar and various volatile compounds, such as hexanal, benzaldehyde, 3-methylbenzaldehyde, 2-butenal, and 4-heptenal. These interactions, including inhibitory effects by *Fusarium* and *Cladosporium*, suggest microbial adaptability to subtropical conditions, potentially influencing fermentation and coffee quality. The sensory analysis showed that the final beverage obtained a score of 80.83 ± 0.39, being classified as a specialty coffee by the Specialty Coffee Association (SCA) metrics. Nonetheless, further enhancements in acidity, body, and aftertaste could lead to a more balanced flavor profile. The findings of this research hold substantial implications for the coffee industry in humid subtropical regions, offering potential strategies to enhance flavor quality and consistency through controlled fermentation practices. Furthermore, this study contributes to the broader understanding of how microbial ecology interplays with environmental factors to influence food and beverage fermentation, a topic of growing interest in the context of climate change and sustainable agriculture.

## 1. Introduction

Coffee is a vital commodity in the global market, serving as a significant export product for numerous developing countries and driving a complex multi-billion-dollar industry [1]. The quality of coffee for export is critically shaped during post-harvest processing, which encompasses key stages such as harvesting, fermentation, and drying [2]. The fermentation step involves the breakdown of sugars and mucilage surrounding the coffee beans, leading to the development of unique aromatic compounds and flavors [3]. In the current coffee processing, open fermentation (natural or dry processing), submerged fermentation (washed or wet processing), carbonic maceration, and induced fermentation are critical techniques that substantially contribute to the development of coffee’s flavor [4]. Among these, open and submerged fermentations are most practiced globally, each influencing the flavor profile in distinct ways [4,5,6].

In the context of climate change, researching new coffee production areas and developing new varieties becomes increasingly crucial. Such initiatives are vital for sustaining the global coffee industry in the face of changing environmental conditions and ensuring the resilience and diversity of coffee crops [1]. The subtropical humid regions, particularly in the state of Santa Catarina, south of Brazil, present a promising frontier for the cultivation of specialty Arabica coffee. This region, not traditionally recognized in the official agroclimatic zoning for *Coffea* species cultivation, has shown remarkable potential due to its unique climatic characteristics. The climatic specificity of the region, characterized by average annual temperatures of 18 to 23 °C, sets a unique stage for coffee fruit development [7]. The region’s historical context of coffee production, combined with recent climatic shifts, suggests a renewed potential for high-quality coffee production. The coffee from this area, especially when grown under organic conditions in banana orchards, has demonstrated sensory qualities that classify it as excellent specialty coffee [7].

In regions with high rainfall, wet fermentation is pivotal in coffee processing, especially for facilitating bean drying [5]. This method involves pulping the coffee cherries and submerging the beans in water-filled tanks, creating an environment conducive to microbial fermentative metabolism. This microbial activity is essential for the efficient removal of pulp and mucilage from the coffee beans. Additionally, it generates ethanol, lactic acid, and various minor compounds, including esters, higher alcohols, aldehydes, and ketones. Coffee beverages derived from wet fermentation exhibit distinct sensory characteristics, such as higher acidity, a lighter body, and a more pronounced aroma compared to dry-processed coffees [8,9]. This disparity can be attributed to the varying metabolic activities of sugars and free amino acids within the seeds, along with intense microbial activity that enhances floral and fruity aromas [2].

In any fermentation process, indigenous microorganisms are of paramount importance. The concept of microbial terroir, which suggests that regional flavors and aromas in fermented products may stem from locally distinct microbial diversity, has garnered attention in the pursuit of capturing the unique geographic characteristics of coffee fermentation in tropical regions. Typically, a variety of yeast species and lactic acid bacteria (LAB) dominate coffee fermentation in countries such as Brazil, Ecuador, Mexico, Australia, Colombia, Honduras, and India [5,10,11,12,13,14,15]. Understanding the composition of the microbiome enables the selection of specific microbial groups to modulate coffee flavors according to regional nuances. Nevertheless, thorough investigation into the microbial terroir of different coffee-producing regions is imperative before implementing practical applications [16].

This study aimed to evaluate the intricate interplay between microbial activity and flavor profile development in coffee fermentation, particularly under the unique conditions of humid subtropical climates. This comprehensive study underscores the pivotal role of microbial communities in shaping the sensory characteristics of coffee, focusing on how varying environmental conditions in these regions influence microbial diversity and activity. By integrating metagenetic and metabolomics techniques, we aim to elucidate the complex biochemical pathways and microbial interactions that occur during coffee processing. This approach not only provides insights into the specific microbial strains that contribute to desirable flavor traits but also highlights the impact of climatic factors on microbial fermentation processes.

## 2. Material and Methods

### 2.1. Area of Study and Sampling Procedure

**Figure 1:**
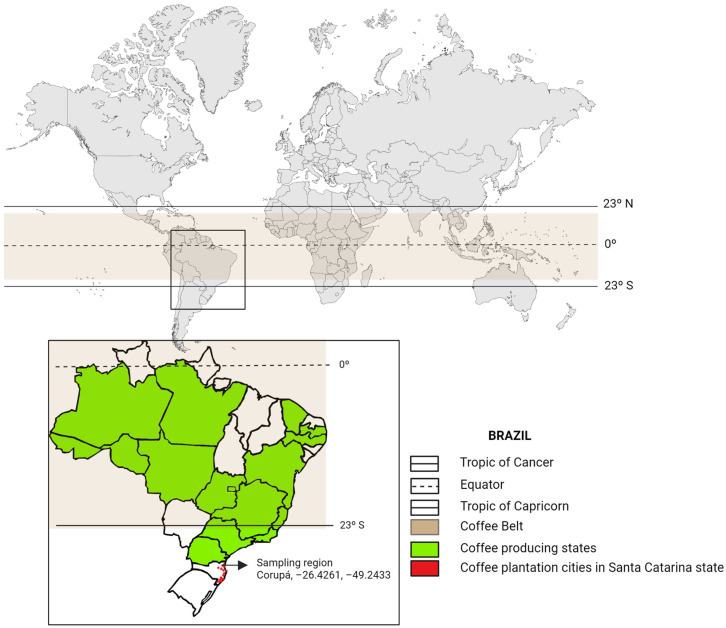
Geographic distribution of coffee processing regions in Brazil with a focus on the sampling area in Santa Catarina state.

Figure 1 illustrates the extensive regions in Brazil dedicated to coffee planting and processing, with a particular focus on the sampling location in Corupá, Santa Catarina State. Situated in a humid subtropical climate zone, this area falls below the well-known coffee production area often referred to as the ‘Coffee Belt’. The coffee cherries (*Coffea arabica* var. Catuaí) were manually harvested and then pulped. Approximately 500 g of cherries were placed into sterile plastic bags, gently squeezed to remove the skins, and subjected to a 24 h wet fermentation (1:1 coffee-to-water ratio). Aliquots of the fraction liquid from the fermenting coffee bean mass were collected at times 0, 12, 17, 20, and 24 h. All samples were collected in triplicate and transported to the Center of Agro-industrial Biotechnology of Paraná (CENBAPAR, Curitiba, Brazil) under refrigeration and kept at −20 °C until further analysis.

### 2.2. Metataxonomic Analysis

Total DNA was extracted from each sample using the DNeasy PowerSoil Pro Kit (Qiagen, Hilden, Germany), following the manufacturer’s recommendations. After extraction, the DNA was quantified using a Nanodrop spectrophotometer (Thermo Fisher Scientific, Waltham, MA, USA). The V3-V4 variable regions of the bacterial 16S rRNA gene were amplified from the total DNA extracted, using primers 341F and 805R. To amplify the ITS region of the fungi, primers 3F and 805R were used [17], both coded with Nextera indices, according to the manufacturer’s instructions (Illumina Inc., San Diego, CA, USA). The samples were sequenced in pairs (2 × 250 bp) using the MiSeq v2 reagent kit and run on a MiSeq platform (Illumina, San Diego, CA, USA). The raw reads obtained during sequencing were analyzed using the parameters of the QIIME (Quantitative Insights into Microbial Ecology) software. Short (<100 bp) and low-quality sequences containing more than one ambiguous base (N) were eliminated. Subsequently, the high-quality reads obtained were aligned with the SILVA database [18] using the UCLUST method [19].

Taxonomic allocation and the generation of operational taxonomic units (OTUs) were carried out using a cut-off point of 97% sequence identity. The number of OTUs obtained in the metagenetic analyses was used to calculate the diversity (Shannon and Simpson) and richness (Chao) indices in the Past program, version 4.03.

### 2.3. Biochemical Analysis of the Fermentation Liquid Fraction

#### 2.3.1. Analysis of Sugar Consumption and Organic Acid Production by High-Performance Liquid Chromatography

Sugars, organic acids, and alcohols were determined by high-performance liquid chromatography (HPLC). The liquid fraction of the fermentation was analyzed on an HPLC system (Agilent Technologies, Waldbronn, Germany) coupled with refractive index (RID) and diode array (DAD) detectors. Compounds were separated using a Hiplex-H column (300 × 7.7 mm) (Bio-Rad, Richmond, CA, USA) with an isocratic mobile phase (4 mmol/L H_2_SO_4_) at a flow rate of 0.5 mL/min for 30 minutes. Quantification of reducing sugars and alcohol was performed using the RID detector, while organic acids were detected by DAD at 210 nm. The concentration of each compound was obtained through analytical curves constructed from standard solutions with known concentrations of lactic, citric, acetic acid, ethanol, glucose, and fructose.

#### 2.3.2. Main Volatile Organic Compounds Identified by Gas Chromatography Coupled to Mass Spectrometry

Volatile organic compounds (VOCs) generated during fermentation were identified using gas chromatography coupled with mass spectrometry (GC/MS). Fermentation samples underwent analysis through Solid Phase Microextraction (SPME), utilizing a DVB/CAR/PDMS Fiber (Supelco Co., Bellefonte, PA, USA). The SPME fiber was exposed for 30 min at 60 °C. Subsequently, compounds were thermally desorbed at 260 °C and directly introduced into the gas chromatograph. The GC system was equipped with a model SH-Rtx-5MS capillary column measuring 30 m × 0.25 mm × 0.25 µm. Temperature settings within the GC included a column oven temperature of 60 °C (held for 2 min), then increased to 220 °C at a rate of 4 °C/min, and finally increased to 240 °C at a rate of 1 °C/min (held for 5 min). The injection temperature was 260 °C, and the detector temperature was 250 °C. Helium was employed as the carrier gas at a flow rate of 1 mL/min, with a column pressure of 57.4 kPa and a split ratio of 1:20. MS analysis ranged from 30 to 250 (*m*/*z*), with an ion source temperature of 250 °C. Volatile compounds were identified by comparing each mass spectrum either with spectra from authentic compounds or with spectra in reference libraries. The relative abundance of each VOC present in the headspace was expressed as peak area multiplied by 10^5^ [5].

### 2.4. Co-Occurrence/Co-Exclusion Analysis

To analyze the statistical interdependencies among various microbial communities and their metabolic products, Spearman’s rank correlation was employed using the statistical software R (version 4.2.3) with the ‘corrplot’ package, which assists in visualizing correlation matrices. The correlation coefficients obtained from this method identified significant positive and negative associations among the studied variables. For a more comprehensive visualization of these relationships, network maps were constructed using the open-source software Gephi (version 0.10.1), which is effective in network analysis and visualization. These maps, depicting the Spearman correlation coefficients as edges, illustrate the complex interactions between microbial species and their influence on coffee flavor profiles during the fermentation process under humid subtropical climate conditions.

### 2.5. Sensory Analysis

To determine the quality of the final beverage, the coffee beans were dried until they reached around 11% humidity, and then a roasting curve was developed to preserve the cellular structure of the beans in a Probat Leogap equipment model Probatino (Curitiba, Brazil). To develop the curve, an initial temperature of 130 °C was used, followed by a gradual increase in temperature allowing for a longer roast (10 min) and a maximum temperature of 220 °C. Cupping was carried out following the recommendations of the Specialty Coffee Association (SCA). Three certified Q-Graders were then asked to describe and score the attributes of aroma, flavor, aftertaste, acidity, body, balance, uniformity, cup cleanliness, sweetness, and overall quality on a scale of 6 to 10, with increments of 0.25.

### 2.6. Statistical Analyses

The data from the analyses of biochemical changes and sensory evaluation were subjected to post hoc comparison of means using the Tukey test. These analyses were carried out using the Statistica program, version 10.0 (Statsoft Inc., Tulsa, OK, USA). The significance level was established using a two-sided *p*-value (<0.05).

## 3. Results and Discussion

### 3.1. Coffea Alpha Diversity during the Fermentation Process

**Figure 2:**
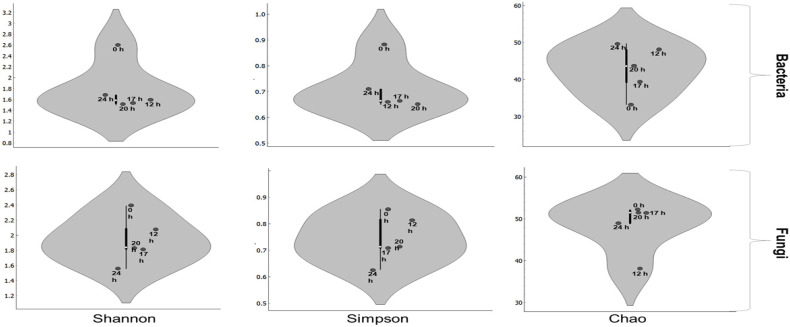
Violin plots of microbial diversity and richness during coffee fermentation in the subtropical zone.

**Figure 3:**
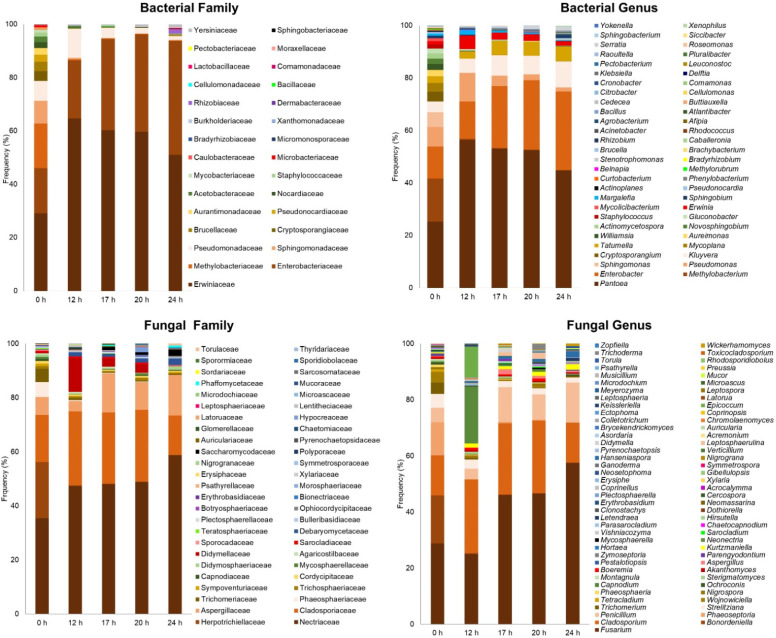
Relative abundance (%) of all microbial taxa at family and genus level identified during coffee fermentation in the subtropical zone.

In the dynamic process of coffee fermentation, microbial communities are essential in shaping the flavor and quality of the final product. This microbiological-driven chemical transformation involves bacteria and fungi, whose presence and activity levels vary significantly throughout the fermentation [2]. These fluctuations are reflected in the changes in microbial diversity and richness, as measured by the Shannon, Simpson, and Chao indices, illustrated in Figure 2. The bacterial and fungal diversity, measured by the Shannon and Simpson Index, gradually decreased throughout fermentation due to the emergence of dominant species, such as *Enterobacter*, *Erwinia*, *Fusarium*, and *Cladosporium* (Figure 3). Initially exhibiting a low Chao index, bacterial richness—indicative of the potential number of different species—experienced a marked increase around the 12 h mark before stabilizing. This significant shift suggests a substantial turnover in bacterial species midway through the fermentation. Conversely, the Chao index for fungi remains relatively stable throughout the fermentation, indicating a consistent richness in fungal species. In summary, fungal diversity and richness seem to experience less pronounced peaks compared to bacterial measurements, possibly indicating a more stable fungal community or less influence from environmental conditions.

### 3.2. Fermentation Diversity and Dynamics

A total of 65.581 bacterial and 37.917 fungi high-quality reads were obtained from the fermentation of coffee beans in the subtropical zone over 24 h. The OTU reads were distributed among 29 bacterial families and 43 fungal families (Figure 3). The Enterobacteriaceae and Erwiniaceae families were the predominant bacterial groups throughout the fermentation. Given that coffee pulp is rich in citrate, pectin, and other complex carbohydrates, bacteria from these families are likely involved in citrate fermentation during the initial stages of the coffee bean fermentation [20], as well as in pectin degradation and extracellular cellulase and protease activities throughout the process [21]. Although this microbial profile is uncommon in coffee fermentations, high-throughput sequencing analyses have shown these bacterial families to be predominant groups in Brazil and Australia [13,17]. Nevertheless, the specific trends for individual families, such as Enterobacteriaceae and Methylobacteriaceae, are somewhat distinct from previous studies, possibly influenced by local conditions or specific fermentation practices. Moreover, other bacterial families such as Methylobacteriaceae, Sphingomonadaceae, and Pseudomonadaceae were present in large populations at the start of the process but experienced a significant decrease throughout fermentation. This trend could be attributed to environmental changes during the fermentation, including shifts in pH, temperature, or resource competition [22,23,24].

The fungal communities during fermentation were predominantly dominated by Nectriaceae, Cladosporiaceae, and, to a lesser extent, Aspergillaceae. The fluctuations and observed changes in these families suggest they possess specific roles or niches within the fermentation ecosystem. Their varying patterns and eventual stabilization or increase might be linked to the evolving conditions during fermentation. This microbial structure, composed mainly of filamentous fungi and enterobacteria, has been observed in coffee fermentations in Central America, South America, and Oceania regions [13,14,17,22,25,26]. However, the role of these microorganisms in fermentation and the mechanisms of interaction are still poorly understood.

At the genus level, the high diversity of bacteria and fungi at the beginning of the coffee fermentation is evident (Figure 3). This initial diversity likely reflects environmental microbial populations that are not specifically adapted to the coffee fermentation environment. As fermentation progresses, conditions such as pH, temperature, and nutrient availability change, favoring organisms that are more suited to the evolving environment of the coffee pulp [4].

*Pantoea* showed an interesting pattern, starting with 25.25% at the beginning of the fermentation and peaking at 56.42% after 12 h, then gradually decreasing to 44.74% by the end. The initial surge and subsequent decline of *Pantoea* suggest its significant but shifting role in coffee fermentation, potentially influencing the early flavor development stages. *Enterobacter* showed a consistent increase from 12.28% at 0 h to 29.99% at 24 h. Other genera of bacteria present in high populations at the start of the fermentation, including *Methylobacterium* (16.35%), *Pseudomonas* (7.45%), and *Sphingomonas* (5.52%), decreased dramatically to minimal levels at 24 h of fermentation. This indicates that these environment-related species are not adapted to coffee fermentation conditions, including nutrient availability and utilization, changes in pH and oxygen levels, competition and succession, and accumulation of metabolic by-products. The increasing trend of *Enterobacter* could be associated with its role in later stages of fermentation, possibly affecting the final flavor profile of the coffee.

Both *Pantoea* and *Enterobacter* are known for their diverse metabolic capabilities, including the production of cellulases, pectinases, and xylanases [27,28]. These enzymes are crucial for breaking down complex carbohydrates found in plant materials, facilitating the release of simpler sugars that are more readily fermentable. *Pantoea*, for instance, has been studied for its role in plant growth promotion and biological control, with a focus on its enzymatic activities related to plant interactions [29]. Similarly, *Enterobacter* species are known for their metabolic versatility, including the ability to degrade plant-derived polysaccharides [30,31]. The metabolic activities of these bacteria can lead to the production of various metabolites during fermentation. These include organic acids, alcohols, and esters, which are key contributors to the flavor profile of fermented coffee [2]. However, the dominance of *Pantoea* and *Enterobacter* presents a notable deviation from the typical microbial profile observed in coffee fermentations in other regions, where LAB are often dominant [5,12]. The prominence of *Pantoea* and *Enterobacter* in this study may reflect a natural adaptation to the specific environmental conditions of subtropical areas, processing methods, or the intrinsic qualities of the coffee beans. Understanding these variations is crucial for tailoring fermentation practices to enhance desired flavor profiles and improve coffee quality. The contrasting microbial dominance observed here could also indicate the potential need for an inoculation process in subtropical coffee fermentation.

At the genus level, *Fusarium*, *Cladosporium*, and *Penicillium* dominated the fermentation process. *Fusarium* showed a significant increase in abundance from 28.80 (0 h) to 57.54% (24 h), while *Cladosporium* and *Penicillium* showed fluctuations during the fermentation. The decline of fungal genera throughout fermentation (e.g.,: *Bonordeniella; Phaeoseptoria*) may be due to changes in environmental conditions during coffee processing, as previously mentioned. *Fusarium* and *Cladosporium*, which exhibit stronger dominance as fermentation progresses, possess enzymatic apparatuses that enable them to utilize complex carbohydrates present in coffee pulp [32,33]. This capability allows them to thrive and outcompete other microorganisms as the fermentation environment becomes more selective. *Fusarium* species are known for their production of a wide array of enzymes, including cellulases and xylanases [34]. *Cladosporium* also possesses enzymes capable of degrading complex polysaccharides [33]. These enzymatic activities enable both *Fusarium* and *Cladosporium* to access nutrients from the coffee pulp, supporting their growth and dominance during fermentation, as recently suggested by Elhalis, Cox, and Zhao and Vale et al. [13,17,26].

### 3.3. Metabolic Profile Changes during Coffee Fermentation

#### 3.3.1. Major Metabolite Changes

**Figure 4:**
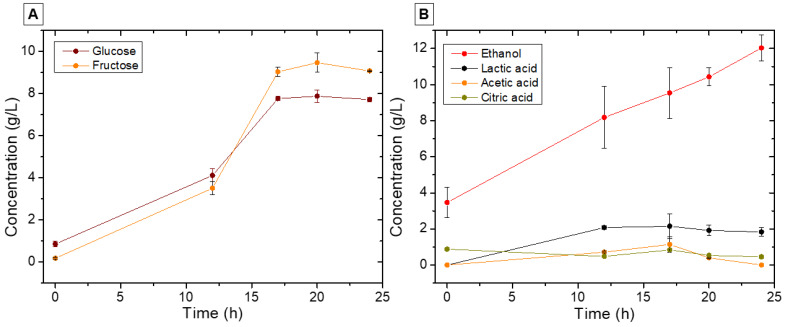
Course of sugar consumption (**A**) and metabolite formation (**B**) during coffee fermentation in the subtropical zone.

Figure 4 presents the concentration changes of various metabolites over time during coffee fermentation. There was a gradual increase in glucose concentration (from 0.85 ± 0.14 to 7.87 ± 0.01 g/L) and fructose (from 0.17 ± 0.03 to 9.47 ± 0.02 g/L) within the initial 20 h of fermentation, attributed to the hydrolysis of complex polysaccharides present in the coffee pulp [4,35]. Following this period, a slight reduction in sugar content was observed, resulting in high levels of glucose (7.71 ± 0.10 g/L) and fructose (9.08 ± 0.01 g/L). This indicated incomplete fermentation, a phenomenon previously observed in spontaneous coffee fermentations with short-duration cycles [36,37], which could result in a lack of depth and complexity in the flavor profile of the coffee. Additionally, there may be a risk of over-fermentation as other opportunistic microbes continue to metabolize the sugars, leading to undesirable flavors and spoilage.

Sugar consumption was directed into ethanol as the primary end metabolite. This compound exhibited a notable increase (*p* < 0.05) over 24 h, beginning at 3.46 ± 0.84 and peaking at 12.03 ± 0.73 g/L. Lactic and acetic acids were identified after 12 hours of fermentation, with peak production (2.14 ± 0.68 g/L and 1.13 ± 0.43 g/L, respectively) achieved by the 17th hour. Ethanol and lactic acid, found in concentrations similar to those observed in prior coffee fermentations, aid in removing the coffee pulp and inhibit the growth of microorganisms that produce off-flavors [5,26,38]. Regarding coffee quality, ethanol acts as a byproduct that contributes to esters and other aromatic compounds’ formation, essential for the coffee’s flavor complexity and depth. Lactic acid, in turn, provides a milder and more pleasant profile than other acids [8,9]. Conversely, the role of acetic acid in fermentation remains uncertain; however, concentrations above 1 g/L may detrimentally impact the final product’s quality [5,26]. Finally, the concentration of citric acid remained relatively stable, starting at 0.88 ± 0.06 g/L, and ending with values around 0.45 ± 0.08 g/L. The stability of this acid during coffee fermentation may be associated with the low presence of yeasts that use this compound as a carbon source [2,39].

#### 3.3.2. Volatile Compounds

**Figure 5:**
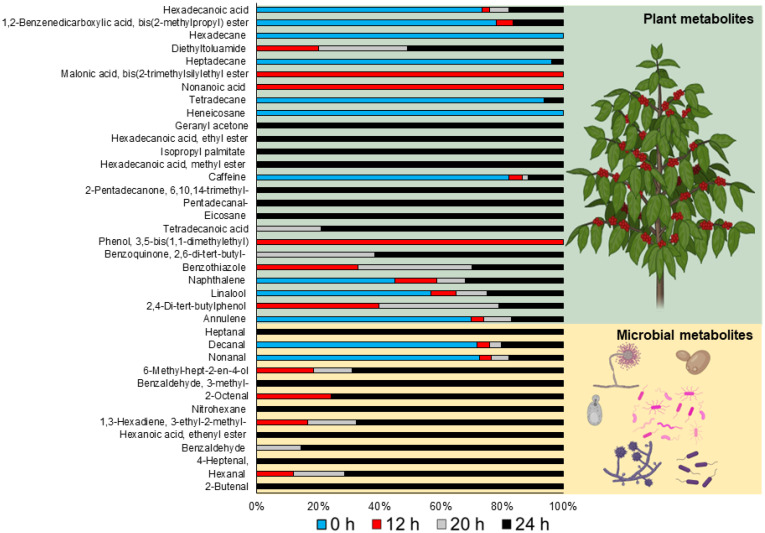
Volatile organic compounds detected during coffee fermentation in the subtropical zone. The differentiation between plant- and microbial-derived based on a comprehensive analysis of studies referenced in [2,17,22,38,40,41,42,43,44,47].

Figure 5 shows the evolving pattern of VOCs over a 24 h fermentation, highlighting the metabolic impact of both plant-derived (25 VOCs) and microbial-derived (13 VOCs) compounds [2,22,24,38,40,41,42,43,44]. The content of each VOC identified throughout the fermentation is also presented in the Supplemental Material, Table S1. The fluctuating levels of volatiles across the time points suggest that microbial activity intensifies as fermentation progresses, with a distinct profile emerging at 24 h. A variety of microbial-associated volatile compounds, including 2-butenal, hexanal, 4-heptanol, hexanoic acid, ethenyl ester, 2-octenal, benzaldehyde, 3-methylbenzaldehyde, and 6-Methyl-hept-2-en-4-ol, were exclusively identified at the end of the fermentation (Figure 5). This could be due to the cumulative effects of enzymatic activities and microbial growth over time, leading to the production of new volatile compounds or the transformation of existing ones [2]. Although members of the Enterobacteriaceae family are often associated with the production of off-flavors in coffee fermentations [15,17,22], *Enterobacter*, *Kluyvera*, and *Pantoea* have been identified as part of the microbiota responsible for flavor formation in fermented foods. This is due to their metabolic versatility, which can contribute to specific esters and alcohol generation [45,46]. It is important to note that the metabolic pathways of these microorganisms are influenced by the coffee bean’s substrate, the fermentation environment, and interactions between microbial communities [11,12,23,46]. The specific metabolites produced during coffee fermentation are often the result of complex microbial consortia rather than individual species [5]. Hence, while these attributions are educated guesses based on typical metabolic products, precise identification requires direct analysis and isolation of compounds produced by each microorganism during fermentation.

From the plant metabolites, it is notable that caffeine remains relatively constant throughout the fermentation, which is expected as caffeine is a stable alkaloid during fermentation. The role of caffeine in coffee plants is multifaceted, influencing not only the taste but also contributing to various ecological functions within the plant [48]. During the processing of coffee, including the fermentation stage, the presence of caffeine remains a prominent characteristic of the beans, contributing significantly to the final product’s sensory profile [40]. Other plant-derived volatiles showed variations that correspond to the breakdown or transformation of plant materials over time. For example, various metabolites present at the beginning of the fermentation process, including hexadecanoic acid, heptadecane, tetradecane, and heneicosane, are components derived from plant material [41,42,43]. Other plant-associated metabolites formed during fermentation may result from plant-aided enzymatic reactions (e.g., geranyl acetone, isopropyl palmitate, pentadecane, and eicosane) or even through the leaching process from the grain to the exterior, like hexadecanoic acid (methyl and ethyl ester), which is one of the main fatty acids found in coffee beans [41,44,49]. It is speculated that the leaching of these compounds may result in a lower integrity of the cell membrane system of the coffee beans. Thus, this cellular damage could affect both the diffusion of volatile compounds from the liquid fraction into the beans and allow chemical components previously compartmentalized in the endosperm to encounter oxidative and hydrolytic enzymes, affecting the quality of the final beverage [50]. However, more studies are needed to analyze these phenomena.

### 3.4. Co-Occurrence/Co-Exclusion Relationships between Genera and Microbial Metabolites

**Figure 6:**
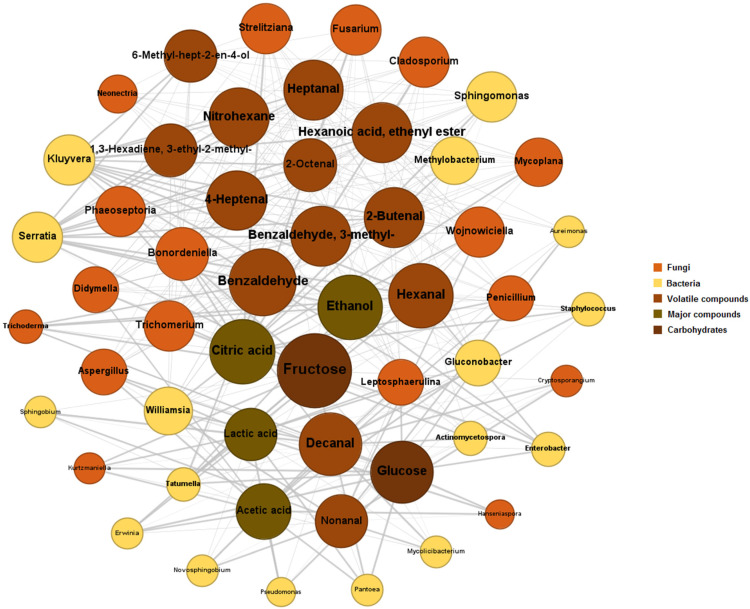
Network map based on the relationships between the main microbiological groups and metabolites generated during the coffee fermentation in the subtropical region. The size of each node is determined by its degree, representing the number of connections it has with other nodes. The thickness of the edges is defined by the weight of the correlation, based on Spearman’s correlation values.

The co-occurrence and co-exclusion relationships between microbial genera and metabolism can be visualized in Figure 6. Using data from an undirected network, 321 connections were found, each representing interactions between microbial species found in the fermentation process and chemical targets with variable weights (Figure 6). It was possible to observe how *Enterobacter*, *Kluyvera*, *Pantoea*, *Serratia*, *Hanseniaspora*, *Pseudomonas*, *Fusarium*, *Cladosporium*, and *Penicillium* interact with various key substances, including glucose, fructose, acetic, lactic, and citric acids, ethanol, benzaldehyde, and 4-heptenal. *Kluyvera* and *Serratia* showed the highest number of connections (12 each) and a significantly positive average weight (0.636), indicating strong interactions with compounds such as benzaldehyde and 4-heptenal. Interestingly, *Fusarium* and *Cladosporium*, both with predominantly negative connections, indicate possible inhibitory effects or chemical competition with other microorganisms during the fermentation. This may also be related to their primary actions on complex carbohydrates present in coffee pulp [26,51], such as pectin and cellulose, which were not measured in this study. *Enterobacter* and *Pseudomonas* displayed a variety of interactions with substances such as decanal and glucose, but with low average weights, suggesting weaker or less influential interactions. Interestingly, 4-heptenal was a common target for a variety of sources, possibly serving as an important intermediary in the production of other key metabolites [2]. These findings demonstrate the adaptability of new microbial groups to subtropical climate conditions, possibly with modifications in metabolic pathways that affect the synthesis of compounds during coffee fermentation. These microbial adaptations to non-tropical conditions can be optimized to maintain or improve coffee quality. This includes the possibility of developing specific starter cultures that can be used to control or direct the fermentation process in favor of desired sensory characteristics [52,53].

### 3.5. Sensory Analysis

**Figure 7:**
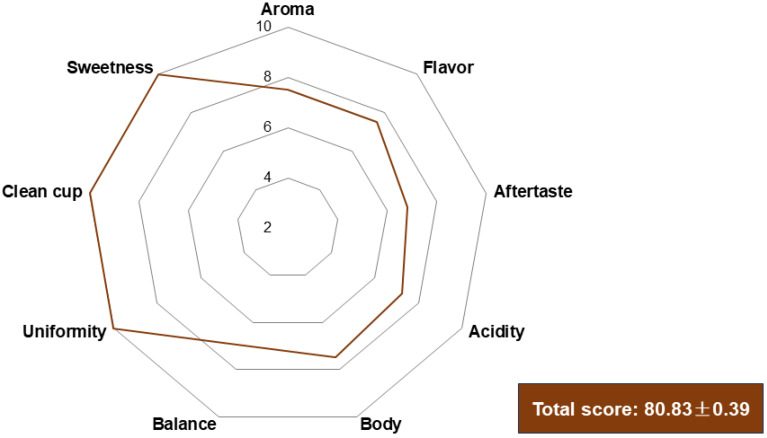
Sensory analysis of the coffee beverage produced from beans fermented by a spontaneous process in the subtropical zone.

The sensory analysis revealed that the final beverage achieved a score of 80.83 ± 0.39, classifying it as a specialty coffee according to SCA metrics (Figure 7). Descriptors noted flavors of butter, caramel, cocoa, malt, dark chocolate, and herbal undertones in the coffee beverage. However, it exhibited low citric acidity, a medium body lacking in cleanliness, and imbalance. The limited sensory complexity may be attributed to the absence of yeasts and LAB during the fermentation process, typically responsible for esters, aldehydes, and lactic acid, crucial for shaping the beverage’s sensory profile [38]. Another hypothesis is that the 24 h fermentation period might have been too short for microbial compounds to fully permeate the beans [17]. Bisso et al. [7] assessed the sensory quality of semi-dry-processed coffee in similar humid subtropical climates. Semi-dry processing involves mechanically depulping coffee fruits and subjecting them directly to sun drying. The authors noted a coffee beverage with more pronounced sugar and caramel-like perceptions, characteristics more commonly associated with dry-processed coffees. This underscores the role of processing methods and microbial activity in shaping the sensory profile of coffee beverages.

## 4. Conclusions

In Santa Catarina’s humid subtropical climate, the microbial community was primarily influenced by *Enterobacter*, *Fusarium*, and *Pantoea*, diverging significantly from the microbial compositions found in typical tropical regions, which are usually dominated by yeast and LAB. Species like *Enterobacter* and *Fusarium* contributed to the formation of distinct flavor compounds such as hexanal, benzaldehyde, 3-methylbenzaldehyde, 2-butenal, and 4-heptenal. These compounds are essential for developing a unique sensory profile that includes fruity and floral notes, distinguishing the coffee produced in this region from others. This resulted in a specific sensory coffee profile that qualifies as specialty coffee.

However, the prevalence of microorganisms such as Enterobacteria and filamentous fungi presents significant challenges. Firstly, a high sugar content was observed at the end of fermentation. This could result in the inefficient production of key metabolites, in contrast to the typical observations in tropical regions where LAB and yeast dominate. In addition, Enterobacteria and filamentous fungi can sometimes be associated with undesirable flavor profiles if not properly managed. Additionally, the resulting beverage exhibited reduced acidity and body, characteristics frequently associated with LAB metabolism, along with flavor and aroma profiles likely influenced by yeast. This scenario highlights the necessity of incorporating specific starter cultures to better regulate these microbial populations, ensuring the consistency and enhancement of the coffee’s desirable flavors.

## Data Availability

The original contributions presented in the study are included in the article/Supplementary Materials, further inquiries can be directed to the corresponding author.

## Supplementary Materials

The following supporting information can be downloaded at: https://www.mdpi.com/article/10.3390/foods13121871/s1, Table S1: Volatile compounds (area × 10^5^) identified in the fermentation liquid fraction.
