# Supplementary material for: Exploring Microbial Influence on Flavor Development during Coffee Processing in Humid Subtropical Climate through Metagenetic–Metabolomics Analysis
Source: Foods. 2024 Jun 14;13(12):1871. doi: 10.3390/foods13121871 (PMC11203001; doi:10.3390/foods13121871)
Supplement: Supplementary file 1 [file foods-13-01871-s001.zip › foods-3029144-supplementary.pdf]

Table S1: Volatile compounds (area × 10^5) identified in the fermentation liquid fraction.

Compounds	Area			
	0 h	12 h	20 h	24 h
2-Butenal	0	0	0	0,739813
Hexanal	0	0,2433991	0,3432578	1,4676949
4-Heptenal,	0	0	0	0,2151519
Benzaldehyde	0	0	0,9514141	5,670388
Hexanoic acid, ethenyl ester	0	0	0	0,4983195
1,3-Hexadiene, 3-ethyl-2-methyl-	0	0,4494029	0,4262436	1,8212942
Nitrohexane	0	0	0	0,7687295
2-Octenal	0	0,296076	0	0,9266417
Benzaldehyde, 3-methyl	0	0	0	1,9241178
6-Methyl-hept-2-en-4-ol	0	0,3459039	0,2341558	1,2919178
Nonanal	7,0178308	0,368653	0,5505816	1,7169341
Decanal	2,034779	0,1147185	0,1127383	0,5695882
Heptanal	0	0	0	0,4712149
Annulene	8,6087335	0,4932988	1,1060459	2,088038
2,4-Di-tert-butylphenol	0	87,230426	85,430755	46,168739
Linalool	3,3398876	0,4840526	0,5881052	1,4676737
Naphthalene	15,047146	4,6044973	3,0848152	10,66634
Benzothiazole	0	1,3521128	1,511754	1,2200345
Benzoquinone, 2,6-di-tert-butyl-	0	0	0,1804738	0,2888365
Phenol, 3,5-bis(1,1-dimethylethyl)	0	0,1273914	0	0
Tetradecanoic acid	0	0	0,1805735	0,6836542
Eicosane	0	0	0	0,1709135
Pentadecanal-	0	0	0	0,2010486
2-Pentadecanone, 6,10,14-trimethyl-	0	0	0	0,401472
Caffeine	18,028774	0,9621367	0,4130519	2,5191576
Hexadecanoic acid, methyl ester	0	0	0	1,0093542
Isopropyl palmitate	0	0	0	0,5815617
Hexadecanoic acid, ethyl ester	0	0	0	1,3904924
Geranyl acetone	0	0	0	0,273059
Heneicosane	1,1550733	0	0	0
Tetradecane	3,0032599	0	0	0,2037612
Nonanoic acid	0	0,1110395	0	0
Malonic acid, bis(2-trimethylsilylethyl ester	0	0,1242323	0	0
Heptadecane	1,6625459	0	0	0,0671151
Diethyltoluamide	0	1,2891043	1,8613085	3,2755533
Hexadecane	1,162428	0	0	0
1,2-Benzenedicarboxylic acid, bis(2-methylpropyl) ester	4,3646102	0,3028776	0	0,9172537
Hexadecanoic acid	34,574932	1,1006777	3,0247261	8,324136
